# Household and community-based approaches to malaria and cholera prevention: A narrative review with case studies from Uganda and Mozambique

**DOI:** 10.1371/journal.pntd.0014186

**Published:** 2026-04-06

**Authors:** Giacomo Guido, Valentina Totaro, Peter Lochoro, Jerry Ictho, Edoardo Occa, Kajal Chhaganlal, Francesco Vladimiro Segala, Giovanni Dall’Oglio, Braunde Francisco Mechendura, Elmano Gomonda, Giovanni Putoto, Federica Penco, Muhammad Asaduzzaman, Emanuele Nicastri, Ferenc Balázs Farkas, Roberta Iatta, Botond Lakatos, Raja Waqar Ali, Annalisa Saracino, Francesco Di Gennaro

**Affiliations:** 1 Clinic of Infectious Diseases, Department of Precision and Regenerative Medicine and Ionian Area, University of Bari, Bari, Italy; 2 Doctors with Africa, CUAMM, Kampala, Uganda; 3 Operational Research Unit, Doctors with Africa CUAMM, Padua, Italy; 4 Department of Research, Faculty of Health Sciences, Universidade Catolica de Mocambique, Beira, Mozambique; 5 Saude familiar e comunitaria, Curso de Medicina Geral, Faculdade de Ciencias de Saude da Universidae Catolica de Mocambique, Beira, Mozambique; 6 Department of Community Medicine and Global Health, Institute of Health and Society, Faculty of Medicine, University of Oslo, Oslo, Norway; 7 Regional Representative of ACGH in Europe, American Society of Tropical Medicine and Hygiene (ASTMH), Arlington, Virginia, United States of America; 8 High Intensity Care Infectious Diseases, National Institute for Infectious Diseases ‘Lazzaro Spallanzani’ IRCCS, Rome, Italy; 9 Institute of Medical Microbiology, Faculty of Medicine, Semmelweis University, Budapest, HungaryPediatric Center, Semmelweis University, Budapest, Hungary; 10 Pediatric Center, Semmelweis University, Budapest, Hungary; 11 Interdisciplinary Department of Medicine, University of Bari, Bari, Italy; 12 Department of Internal Medicine and Hematology, Departmental Group of Infectious Diseases, Semmelweis University, Budapest, Hungary; International Centre for Genetic Engineering and Biotechnology, INDIA

## Abstract

**Background:**

Housing conditions play a fundamental role in infectious diseases transmission, particularly in endemic regions. Structural modifications to homes—such as screened windows and doors, sealed eaves, elevated flooring, and insecticide-treated barriers—are increasingly recognized as key strategies for reducing mosquito entry and, consequently, malaria risk. Similarly, improved latrine, proper waste disposal, and the possibility to hand wash can help to reduce the spread of waterborne diseases such as cholera. However, their effectiveness and feasibility vary across geographical and socio-economic settings. This study examines the impact of housing-based interventions on malaria and cholera prevention through two case studies conducted in Uganda and Mozambique.

**Methods:**

A literature review was conducted alongside case studies from Uganda and Mozambique, where housing interventions for endemic infectious diseases prevention have been implemented. Data were collected from scientific literature, local health reports, and field observations.

**Results:**

The combination of multiple preventive measures at the community level, including both structural modifications to housing and changes in domestic behavior, holds great potential in combating various endemic diseases in low-income countries, such as malaria and cholera. These integrated strategies are generally low-cost, community-acceptable, and easily implementable, and can complement existing large-scale control programs.

**Conclusion:**

House-based interventions represent a sustainable and complementary strategy for malaria and cholera control in endemic regions. However, their successful implementation depends on affordability, cultural adaptation, and integration with existing control programs. Future research should focus on scaling up these interventions and evaluating their long-term impact on vector- and water-borne diseases transmission in endemic and low-resource settings.

## Author summary

The spread of infectious diseases like malaria and cholera continues despite existing preventive measures; indeed, it’s worsening within a global context of climate change, disproportionately impacting low-resource populations. Adopting specific individual behaviors and making small house modifications, such as adding screens to windows or improving toilets and waste disposal, can significantly contribute to preventing these vector- and water-borne infections. We describe two low-cost structural and behavioral implementation models at the household level that integrate various useful measures for the prevention of malaria and cholera in endemic countries like Uganda and Mozambique, respectively. Evidence reveals the intricate challenges hindering malaria and cholera prevention, encompassing socioeconomic disadvantages, established cultural viewpoints, and the broad effects of environmental and climate change. Ultimately, the analysis of the determinants of human behavior and robust community engagement in prevention strategies are fundamental for successful implementations.

## Introduction

The global landscape of infectious diseases is continually evolving. The spread of vector-borne, waterborne, foodborne, and airborne diseases is strongly influenced by climate change and climate-related disasters [1], uncontrolled urbanization [2], the intensification of international travel [3], intensive agriculture, and industrial livestock farming [4,5]. Additionally, frequent and prolonged conflicts play a significant role [6,7]. Human activities, therefore, exert a profound impact—sometimes not immediately apparent—on the health of ecosystems and, ultimately, on human well-being [8]. The greatest burden of these diseases disproportionately affects low-income countries and the most vulnerable populations, including marginalized people living in rural areas and internally displaced peoples (IDPs) as well as refugees. In the poorest settings, diseases such as malaria and cholera remain leading causes of morbidity and mortality [9,10].

Globally, in 2023, the number of malaria cases was estimated at 263 million, with an incidence rate of 60.4 cases per 1,000 individuals at risk. This represents an increasing trend compared to the previous year. In contrast, malaria-related mortality has declined, with an estimated 597,000 global deaths in 2023 and a mortality rate of 13.7 per 100,000 population.

The WHO African region continues to bear the highest burden of the disease, accounting for an estimated 94% of global malaria cases and 95% of malaria-related deaths in 2023.

In 2023, malaria-endemic countries experienced a disproportionate burden of conflicts and climatic disasters, creating ideal conditions to malaria spread [9]. Over the decades, areas previously unsuitable for the transmission of *Plasmodium falciparum* and *Plasmodium vivax* have become environmentally conducive to their spread [1]. Climate-change driven rising temperatures and altered rainfall patterns, have contributed to the resurgence of malaria in some locations [9]. Given rapid and poorly planned urbanization, the spread of an invasive mosquito species, *Anopheles (An.) stephensi,* in African cities is posing another threat in malaria epidemiology as well as the emergence of antimalarial resistance [9]. In addition, reduced healthcare access during the COVID-19 pandemic exacerbated malaria outcomes, resulting in heightened global incidence and mortality [11]. Furthermore, recent foreign aid cuts are undermining efforts to control and eliminate malaria with serious consequences for communities [12].

In the last 20 years, cholera has shown a consistent global decrease in incidence, largely due to targeted control programs aimed at improving hygiene practices, commonly referred to as Water, Sanitation, and Hygiene (WASH) initiatives [13]. However, since 2021 an increase in cholera cases and multiple outbreaks has been recorded, with a further peak in 2023, consequently leading to a rise in mortality rates. In Mozambique, specifically, approximately 28,000 cases were recorded in April 2023, a tenfold increase compared with February 2023 data [14].

The recent cholera epidemics, along with other waterborne diseases (WBD), can be attributed to three main factors: climate change, conflicts and humanitarian crises, vaccine shortage, and underinvestment in national water and sanitation services [15].

The incidence of cholera and other causes of acute watery diarrhea (AWD) is influenced by water temperature, salinity, and extreme weather events, which increase disease risk by triggering bacterial growth and damaging water sanitation infrastructure, leading to greater human exposure. Such damage not only creates barriers to healthcare access but also disrupts supply chains for essential commodities and medicines, further complicating cholera outbreak response and management [16].

**Fig 1:**
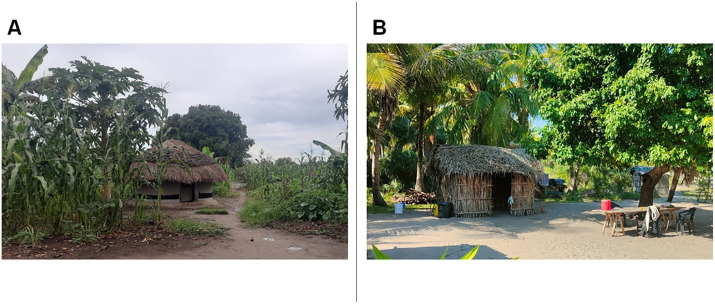
Traditional houses. **(A)** Uganda. **(B)** Mozambique.

Large-scale disease control programs in low-income regions are often hindered by weak governance, fragile health systems, and ongoing conflicts or natural disasters. In such contexts, small-scale, community-led initiatives based on the adoption of simple behaviors and household modification can serve as effective tools for preventing and controlling the transmission of infectious diseases [17]. Various aspects of substandard housing (Fig 1), such as rudimentary materials, overcrowding, lack of access to safe water, and inadequate sanitation, have long been identified as significant risk factors for diseases like diarrhea, respiratory infections, and malaria [18–20]. Inadequate housing can affect individuals’ health through multiple mechanisms, including increased exposure to disease vectors in the absence of mosquito nets, indoor air pollution and burn injuries associated with the use of solid fuels for cooking, heating, and lighting, thermal stress due to poor ventilation, as well as heightened vulnerability to robbery, physical assault, landslides, floods, and storms in the case of fragile housing structures [18]. A 2020 cross-sectional analysis conducted to examine the relationship between housing conditions and child health across sub-Saharan Africa found that unimproved homes, defined as those lacking adequate water and sanitation facilities, providing insufficient living space, and constructed with unfinished materials (e.g., earth, sand, dung, or palm flooring), were associated with an increased likelihood of malaria, diarrhea, stunting, wasting, underweight, and anemia [19]. Additional evidence links acute respiratory infections to indoor air pollution, poor ventilation, and overcrowding [21,22].

Ensuring good-quality housing is essential in reducing the burden of these illnesses, improving health outcomes contributing to the achievement of the Sustainable Development Goals (SDGs) [23]. Similarly, personal behavior plays a crucial role in preventing the spread of infectious diseases, particularly during an outbreak, and adherence to recommended behaviors is essential for the success of infection control interventions [24–26].

This study seeks to assess the impact, relevance, and feasibility of housing and behavioral measures for the prevention of malaria and cholera, and to present two integrated implementation models, one from Uganda for malaria and one from Mozambique for cholera, as illustrative examples of community-level disease prevention strategies.

## Methods

This work adopted a narrative literature review design, complemented by case studies from Uganda and Mozambique. The review aimed to synthesize evidence on household and community-level interventions for malaria and cholera prevention, while the country examples were used to illustrate practical implementation experiences and contextual factors influencing effectiveness.

A comprehensive narrative review of the scientific and grey literature was conducted to identify studies published between 2010 and 2024 on household and community-level interventions for malaria and cholera prevention. Searches were performed in PubMed, Scopus, and Google Scholar using combinations of keywords such as “malaria prevention”, “cholera prevention”, “household”, “community”, “housing improvement” and “behavioral interventions”. Additional relevant reports were identified through manual searches of World Health Organization (WHO) and national health ministry websites. The review focused on studies conducted in low- and middle-income countries, particularly in sub-Saharan Africa, and findings were synthesized thematically to highlight common patterns, implementation challenges, and lessons learned relevant to Uganda and Mozambique.

For the case studies, Uganda and Mozambique were selected due to the NGO ‘Doctors with Africa CUAMM’ (University College of Aspirants for Missionary Medicine) experiences in community interventions to prevent infectious diseases. These countries also represent distinct geographical, socioeconomic, and policy contexts, allowing for comparative insights into intervention effectiveness. We provide a description of two implementation projects addressing housing and behavioral interventions, designed in accordance with evidence from the literature and informed by an evaluation of the local context. The description focused on identifying barriers and facilitators to implementation—such as cost, cultural acceptability, and policy support—and assessing the scalability and sustainability of housing and behavioral modifications as malaria and cholera prevention strategies.

## Results

### Uganda case study

#### Issues in malaria prevention.

Interventions to control malaria include chemoprevention for preschool children and pregnant women, long-lasting insecticide-treated nets, indoor residual spraying and outdoor space spraying, finally active and passive immunization development [27]. However, despite the consistent application of existing malaria control tools, the Global Technical Strategy for Malaria 2016–2030 (GTS) and the Sustainable Development Goal (SDG) targets for malaria morbidity and mortality in 2025 and 2030 are unlikely to be achieved [28,29].

Many interventions, especially on vector control, however, are closely linked to human behavior and the surrounding environment of the main dwelling.

Globally, household malaria prevention has primarily relied on the use of insecticide-treated bed nets (ITNs) and indoor residual spraying (IRS), which, however, cover less than 50% of the population [9].

The literature suggests that combining multiple malaria prevention interventions is more effective than using a single practice in reducing malaria incidence and prevalence. Nevertheless, in most cases, the combination involves only two methods, primarily ITNs and IRS [30,31].

A comprehensive approach combining different household prevention methods is not yet common practice [32,33] and the most commonly known preventive measure, often the only one used, is sleeping under an insecticide-treated bed net [34,35].

Despite the Uganda Malaria Indicator Survey 2018–2019 reports that 83% of households own at least one ITN and 10% of all households received IRS, the percentage of use is lower (59%) [36]**.** As far as government-supplied nets, the main reported reason is to save them for future use [37]**.** Net care attitudes vary: in many cases, nets are damaged by children or rodents and are rarely repaired [38–40]**.** while old nets that have lost their insecticide efficacy are still being used [41]**.**

Consequently, human behavior can significantly impact the efficacy of established preventive measures.

Environmental management is seldom included in prevention practices, such as the use of window and door screens, traditional plants, and the removal of potential larval breeding sites [42,43].

Numerous studies across sub-Saharan Africa have associated improved housing – specifically screening of windows and doors, finished materials for roofs, walls, and floors and closed eaves instead of mud and thatched traditional house – with decreased mosquito density [44,45] and malaria incidence [46–48] lowing malaria burden at the community level. Consistent with the systematic review by Fox et al. (2022), the implementation of screened homes significantly reduced indoor mosquito density by 37% during nocturnal hours. Moreover, inhabiting a modified residence correlated with a 32% lower likelihood of blood parasite infection and a 30% reduction in the prevalence of anemia [45]. In the end, modifying houses can provide extra protection in addition to the benefits of using ITNs and IRS [49,50].

The surrounding outdoor environment, where mosquito breeding sites are located [51], should also be considered for malaria prevention [52,53]. In much of sub-Saharan Africa, dwellings are interspersed with small-scale agriculture being significantly correlated with local breeding sites, risk of malaria and mosquitoes susceptibility to ITNs [54]. Many studies extensively documented the association between the presence of irrigated crops, especially rice and cereals, and proximity to wetlands with a high *Anopheles* exposure and malaria transmission, likely due to increased mosquito breeding sites [55–58]. In addition, maize pollen, which is abundant during the wet season on the water surface near plantations, seems to be an excellent food for the mosquito larvae [59]. Tree holes, particularly those of acacia, avocado and mango trees, can also provide breeding sites [60].

Plants, such as *Eucalyptus* spp., *Ocimum* spp. and *Cymbopogon* spp. [61], have traditionally been used by humans for the treatment of malaria [62] as well as for protection against mosquitoes. The extraction of essential oils [63–65], burning the plants [66], consuming them [67], hanging them inside the house, scattering their leaves on the floor [68], and planting them near houses [67] seem to be good practices against malaria. In particular, the latter has proven to be effective in reducing the indoor population of malaria vectors [69]. However, traditional herbal remedies have numerous limitations, especially when ingested. For example, the use of non-pharmaceutical forms of artemisia is often ineffective and could accelerate the development and spread of artemisinin resistance, which is currently one of the four global threats to malaria control [70].

Other simple, known and no-cost environmental control measures include covering water containers or disposing of empty containers to avoid water accumulation [71], removing excess vegetation around homes and closing windows and doors early in the evening [72].

Several control strategies can still be implemented and integrated at household level to reduce malaria burden [73].

The 2024 World Malaria Report ranks Uganda as the third-highest contributor to the global malaria burden [9]. In 2023, Uganda recorded an estimated 12.6 million malaria cases and nearly 16,000 deaths [9]. The country experiences an annual economic loss of more than $500 million due to malaria [74].

The 2021–2025 Uganda Malaria Reduction and Elimination Strategic Plan aims to reduce malaria morbidity by 50% and malaria-related mortality by 75% from 2019 levels by 2025 [75].

To achieve this, the Government of Uganda advocates to decentralize malaria reduction and elimination plans by implementing research and mass action initiatives across all sectors, communities, and households [76,77]. The Malaria Actions Against Malaria (MAAM) approach supports integrated preventive measures and proposes a smart malaria model to achieve a malaria-free home, village, parish, sub-county, county/constituency, district, region, and Uganda [78,79].

This study was conducted with the aim of increasing community awareness of the integrated approach to malaria prevention and promoting the creation of ‘Malaria Smart Homes’—places where a series of measures are implemented to limit the proliferation of *Anopheles* mosquitoes.

#### The “Malaria smart home model” - Uganda.

Responding to the Ugandan government’s appeal, the NGO ‘Doctors with Africa CUAMM’ proposed applying the “Smart Home” model to villages in Oyam District, Lango Sub-region, Northen Uganda. A total of four sub-counties in Oyam District were involved: Aber, Acaba, Icene, and Myene.

Lango is classified among the medium transmission intensity regions of Uganda, with malaria incidence ranging between 251 and 450 cases per 1,000 population [80,81].

Oyam District was selected due to its high malaria incidence and related deaths in recent years, which exceeded the expected epidemic threshold [36,82].

According to the last Uganda Bureau of Statistic report, Oyam District hosts 76,536 households with a total population of 383,644 people. The majority of the population (91.54%) resides in rural areas with subsistence farming as a main source of livelihood. 71.5% of households is living in temporary dwelling units, those built using temporary materials for the roof, wall and floor. (Fig 1A) A large portion of the population faces geographical difficulties in accessing basic services, including healthcare and education [83].

*Plasmodium falciparum* is the main parasite species accounting for 97% of malaria infection [36], and *An. funestus s.l.* and *An. gambiae s.l.* are the most widespread vectors in Uganda [84]. The *An. gambiae* species complex is known for its preference for indoor blood meals (endophagy), feeding on humans (anthropophily), and resting indoors (endophily) [85].

The first step was to conduct education and awareness programs in the villages, utilizing CUAMM social workers, local health operators, and community leaders to disseminate information about malaria, including the route of transmission, symptoms, and effective prevention strategies.

Based on mosquito behavioral patterns [86,87], daily activities in the village and evidence supporting the efficacy of household interventions [43,49,69], the selection criteria prioritized readily implementable and sustainable family-level strategies from the Ugandan government’s proposed national malaria elimination plan [76,77].

**Fig 2:**
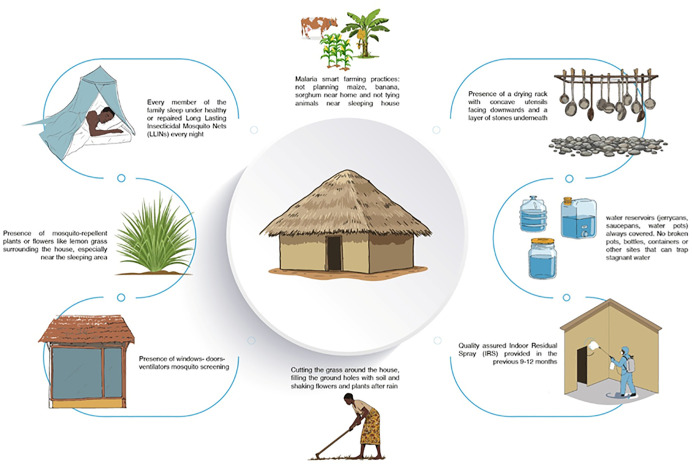
Malaria Smart Home Model.

The following criteria were chosen to define a “Smart Home” household (Fig 2):

- every member of the family sleep under healthy or repaired Long Lasting Insecticidal Mosquito Nets (LLINs) every night;
- presence of windows/doors/ventilators mosquito screening;
- closing windows/doors by 6pm;
- quality assured Indoor Residual Spray provided in the previous 9–12 months;
- water reservoirs (jerrycans, saucepans, water pots) always covered. No broken pots, bottles, containers or other sites that can trap stagnant water;
- presence of a drying rack with concave utensils facing downwards and a layer of stones underneath;
- malaria smart farming practices: not planting maize, banana, sorghum near home and not tying animals near the sleeping house;
- presence of mosquito-repellent plants or flowers like lemon grass surrounding the house, especially near the sleeping area;
- cutting the grass around the house, filling the ground holes with soil and shaking flowers and plants after rain.

The use of each measure in terms of prevention is explained in Table 1

**Table 1:** *Anopheles* mosquito habits, “Malaria Smart Home” criteria and their effectiveness in terms of malaria prevention.

	Anopheles Mosquito Habits [86,87]	Implementation	Effectiveness
**Indoor activities**	Adult female *Anopheles* mosquitoes usually bite people at night. They require a blood meal for egg development.	**1. Sleeping under LLINs every night**	Protect all family members from night bites.
	*Anopheles* mosquitoes rest indoors during the daytime attracted to dark, sheltered areas.	**2. Windows/doors/ventilators mosquito screening**	Preventing mosquitoes from entering the house.
	Adult female *Anopheles* mosquitoes mostly bite between sunset and sunrise (6 pm to 6 am).	**3. Closing windows/doors by 6pm**	Keeping mosquitoes out of the house during times when they are most active.
	*Anopheles* mosquitoes rest indoors during the daytime attracted to dark, sheltered areas.	**4. Quality assured IRS**	Killing adult mosquitoes coming in contact with treated surfaces preventing mosquitoes from resting on the walls of the house
**Outdoor activities**	Adult female *Anopheles* mosquitoes lay eggs on the still water’s surface, where the larvae hatch and mature. Eggs do not survive drying.	**5. Covering water reservoirs and dispose of any unused containers**	Avoid providing water surfaces where mosquitoes can lay eggs.
	Adult female *Anopheles* mosquitoes lay eggs on the water’s surface. Eggs do not survive drying. The larvae hatch and mature in the water.	**6. Drying rack with a layer of stones underneath and drying tools upside down**	Preventing the creation of breeding sites: dry tools to avoid small water collections from forming and ensure runoff water drains properly, preventing its accumulation on the ground below.
	Breeding sites include swamps, rice fields, grassy ponds, tree trenches, irrigation canals, ditches, the edges of rivers, and small temporary rain puddles.	**7. Malaria smart farming practices**	Avoiding breeding sites and the circulation of mosquitoes around the house.
	Adult *Anopheles* mosquitoes are thought to typically disperse a few hundred meters within villages	**8. Planting mosquito-repellent plants or flowers**	Keeping mosquitoes away from the home area
	Some *Anopheles* species can rest outdoors in suitable resting places, such as holes, animal sheds, and dense vegetation.	**9. Cutting grass, filling holes, shaking plants**	Disturbing mosquitoes’ environment and reducing their circulation around the house.

**Fig 3:**
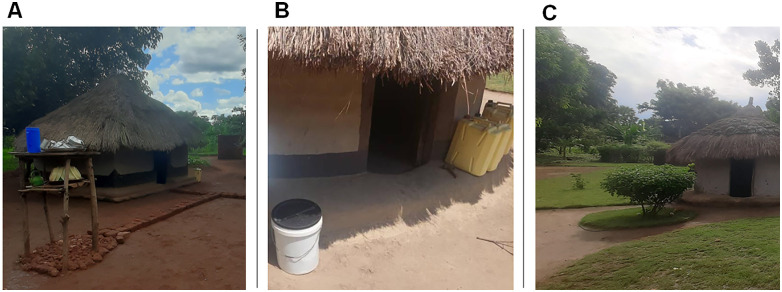
Representative “Malaria Smart Home” implementations in Uganda. **(A)** Drying rack. **(B)** Covered water reservoirs. **(C)** Cut grass and mosquito-repellent plants near the house.

A “Malaria Smart Home” household is a place characterized by all the malaria-safe behaviours and housing interventions included in the list and can maintain them over time. In this regard, quarterly monitoring of all households in the involved villages was conducted to assess the implementation of all measures and the achievement of ‘Smart Home’ status. Additionally, it aimed to ensure that households already designated as ‘Smart Homes’ maintained their status over time. Fig 3 presents representative photographs from the field.

### Mozambique case study

#### Drivers of cholera spread in Mozambique and behavioral approach.

In Mozambique, since 2019, within a relatively short period, the country has experienced multiple extreme weather events, ranging from Cyclone Idai to Cyclone Freddy and Cyclone Chido, highlighting the country’s geographical vulnerability to tropical cyclones, particularly in the northern Cabo Delgado region [88,89]. As of early January 2025, United Nations High Comissioner for Refugees (UNHCR) data reports over 700,000 IDPs in the country [16]. However, the impact of these events extends beyond destruction and environmental degradation, also leading to reduced food security, stagnating economic growth, and worsening public health conditions.

The period 2019–2021 was not only marked by severe climate crises in Mozambique but also by an intensification of the internal conflict with non-state armed groups (NSAGs) leading to widespread insecurity along with an exacerbated number of IDPs [90]. Displacement and overcrowding, coupled with poor hygiene conditions, inevitably increased the risk of transmission of WBD [91]. Additionally, the destruction of healthcare facilities and the difficulty in accessing medical services further aggravated the crisis.

In this context, extreme climate changes inevitably necessitate massive investments in restoring access to safe drinking water and rebuilding villages. However, such initiatives are not easily implementable in a conflict-affected area, particularly in a low-income country like Mozambique, which ranks seventh among the world’s poorest nations in terms of gross national income (GNI) per capita [92]. Moreover, internal socio-economic disparities further complicate the country’s ability to effectively address these challenges.

All these conditions create a scenario in which overcrowding coincides with the presence of stagnant water contaminated with *Vibrio cholerae*, while the lack of access to safe drinking water forces the population to use contaminated water for hygiene, thereby facilitating the spread of cholera [10,93]. Simultaneously, the use of contaminated water for agricultural purposes contributes to foodborne transmission, further exacerbating the spread of infection. These risks are compounded by the high prevalence of malnutrition in overcrowded settings and among IDPs, which can lead to more severe disease outcomes [94].

The resurgence of cholera underscores the need for sustained investments in climate-resilient WASH infrastructure and public health systems. Future perspectives highlight the importance of addressing socioeconomic disparities and enhancing global coordination to prevent outbreaks. However, in the absence of adequate and robust control policies to mitigate the impact of cholera outbreaks and achieve the 2030 Cholera Elimination Goals [13], a viable and easily implementable alternative lies in proactive strategies. These include early detection but, most importantly, community engagement, with interventions aimed at enhancing preparedness, such as social behavior change initiatives.

The social structure and relationships with formal and informal leaders represent a significant determinant: the low involvement detected by the authorities in the dissemination of preventive campaigns impacts the stimulus to the population, limiting their access to key information and social gratification models to follow.

The recurrence of epidemics impacts the cultural and psychological determinants of family aggregates; the ability and possibility for families to objectively reduce the problem thanks to individual decisions and choices is limited. The low awareness in “self-effectiveness” is a key element in taking on responsibilities at the individual and family level.

In light of these perspectives, it becomes crucial to design effective and sustainable prevention programs, to define a strategy that considers behavioral determinants as an essential element from the design of the intervention [95].

In this context, we point out from a medical anthropology perspective, how the epistemological model of infectious disease sciences must be integrated with a reading that also considers factors peculiar to local culture, such as the nuanced understanding of the biological cause-effect model and therefore of the causality of behavior and risk of infection, or how the relational and intimate sphere contributes to the justification and understanding of disease as relevant variables in the field of prevention and public health [96].

#### The “Familia Modelo Model” for cholera – Mozambique.

Based on mixed-methods research conducted in Cabo Delgado within the framework of social behavior change programs, both qualitative and quantitative data indicate that many of the key factors driving the spread and recurrence of cholera epidemics in the province are behavioral. These behaviors are often shaped by collective psychology as well as deep-rooted sociocultural norms and practices [97].

Between September 2022 and May 2024, UNICEF and Doctors with Africa CUAMM carried out the project titled “Support the Adoption of Key Behaviors to Prevent COVID-19, Cholera, Malaria, Malnutrition and Improve Access to Services in Cabo Delgado.” The project focused on encouraging preventive behaviors among rural populations in six districts of Cabo Delgado Province. As a component of this effort, a Social and Behaviour Change (SBC) intervention known as “Família Modelo” was introduced. This intervention aimed to enhance the uptake of fourteen practical, low-cost behaviors intended to reduce vulnerability to climate-sensitive outbreaks [98]. The project directly contributed to the Ministry of Health’s efforts to curb the spread of acute watery diarrhea (AWD) and cholera by encouraging the broad adoption of critical WASH practices, including regular handwashing, safe water treatment, and appropriate waste disposal. Additionally, it supports the WHO's recommendation to incorporate behavioral science into public health strategies, with the goal of gaining deeper insight into the drivers of disease and developing more effective health interventions [99].

**Fig 4:**
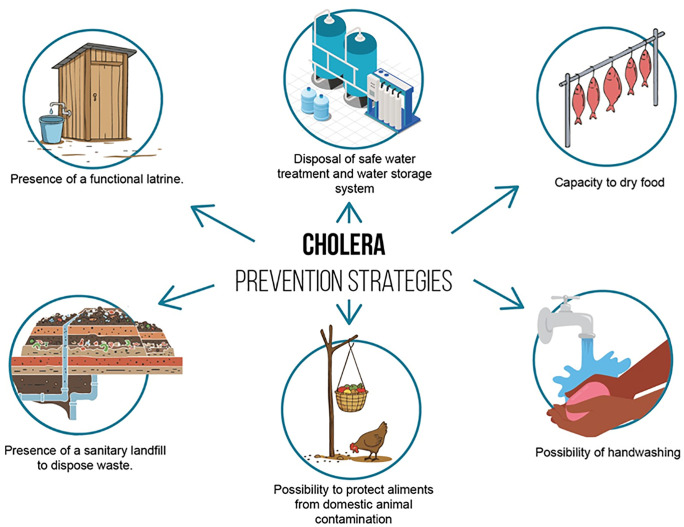
The “Familia Modelo Model”.

The intervention encompassed not only cholera control measures but also practices aimed at controlling vector-borne and waterborne diseases (Table 2). Specifically, the intervention centered on seven key adaptation practices, illustrated in Fig 4, each serving as an indicator of household resilience to infectious disease outbreaks:

**Table 2:** Practices implemented in Familia Modelo.

Intervention	Effects on Cholera and WBDs	Effects on VBDs
**1. Presence of insecticide treated mosquito nets**	**Low** Reduction in secondary infections that may weaken individuals	**High** Protection from vectors (e.g., *Anopheles* and *Aedes*) with consequent reduction of pathogen transmission
**2. Presence of a functional latrine**	**High** Reduction of fecal contamination of water sources	**Intermediate** Reduction of the accumulation of stagnant water and organic matter, which could otherwise serve as breeding sites for vectors
**3. Presence of a sanitary landfill to dispose waste**	**High** Reduction of the accumulation of organic waste, which can contaminate drinking water sources and promote the spread of bacterial and viral infections	**Intermediate** Elimination of breeding sites for vectors
**4. Capacity to dry food**	**Intermediate** Reduction of the growth of moisture-dependent pathogens and parasites	**Low** Proper food preservation reduces insect attraction, lowering the risk of contamination from vectors
**5. Possibility to protect aliments from domestic animal contamination (i.e.,: availability of a pylon stick)**	**High** Prevention of contamination by fecal matter and zoonotic pathogens	**Low** Reduction of the presence of vectors that act as mechanical vectors of enteric pathogens.
**6. Possibility of handwashing**	**High** Drastically reduction in fecal-oral route transmission of pathogens	**Low** Reduction of the risk of indirect transmission of pathogens carried by vectors
**7. Disposal of safe water treatment and water storage system**	**High** Elimination of WBDs	**Intermediate** Prevention of laying eggs from *Aedes* mosquitoes in open containers

- Presence of insecticide treated mosquito nets
- Presence of a functional latrine
- Presence of a sanitary landfill to dispose waste
- Capacity to dry food
- Possibility to protect aliments from domestic animal contamination (i.e.,: availability of a pylon stick)
- Possibility of handwashing
- Disposal of safe water treatment and water storage system

**Fig 5:**
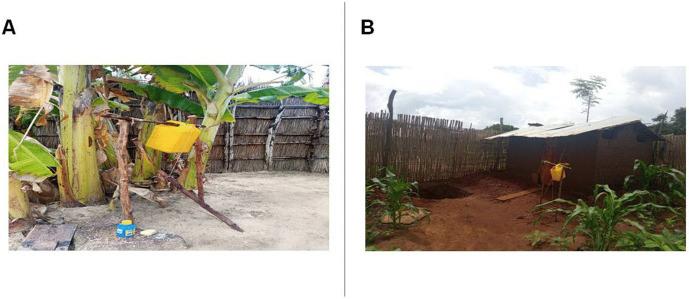
Representative “Familia Modelo” interventions in Mozambique. **(A)** Hand washing tool. **(B)** Tidy rubbish pit.

Each household was evaluated using a preparedness score ranging from 0 (complete unpreparedness) to 7 (high preparedness), based on the presence of these adaptation strategies. The intervention was implemented across six districts in Cabo Delgado province, between October 2022 and December 2023, relying on community health workers who conducted weekly household visits to provide education, distribute materials, and monitor the uptake of key practices [89]. Selected field photographs are presented in Fig 5.

In this context, a cohort study involving 1,602 family units demonstrated marked improvements in key behaviors. Specifically, handwashing increased by 57.6%, and safe water treatment and storage rose by 49.7% (article under review). Furthermore, a cross-sectional study conducted within the same population revealed that both behaviors were significantly associated with a lower likelihood of reporting a recent household episode of acute watery diarrhea (AWD). The adjusted odds ratios were 0.47 (95% CI: 0.29–0.78) for the presence of handwashing facilities and 0.31 (95% CI: 0.18–0.52) for access to safe water [89]. However, we were not able to directly demonstrate a causal effect of the SBC intervention on the incidence of AWD.

This initiative was developed and promoted within the framework of a broader social behavioral change program led by Doctors with Africa CUAMM and UNICEF Mozambique, piloting the national strategy for communicable disease prevention in humanitarian settings, integrated as well by the Mozambican Minister of Health.

## Discussion

Our findings contribute to a more integrated understanding of the role of housing and household-level interventions in the prevention of malaria and cholera. By combining evidence from the literature with real-world experiences from Uganda and Mozambique, this study highlights how household infrastructure, sanitation, and behavioral practices interact with community-level factors to shape disease risk.

The analysis underscores that housing interventions—such as improved water storage, waste management, and vector-proof housing—cannot be addressed in isolation, but require alignment with local social dynamics, community engagement, and health education.

This synthesis helps bridge the gap between research and implementation, advancing the understanding of how household-focused strategies can complement public health and vector control programs in low-resource settings.

While the study offers useful insights into housing and behavioral interventions for disease prevention, several limitations should be acknowledged, primarily related to its descriptive nature and the absence of primary data and quantitative analysis.

Housing modifications for malaria prevention have shown promising results in different settings, as demonstrated in Uganda and Mozambique. As reported in previous experiences, such implementations were well accepted by local populations, with families expressing satisfaction in receiving housing improvements and demonstrating great care in maintaining them [33]. These kinds of housing improvements not only contributed to a malaria-free environment [100,101] but also enhanced the aesthetic, organizational, and hygienic conditions of households, setting an example for the entire community. Despite the effectiveness of such interventions, the adoption of malaria-preventive measures is influenced by behavioral determinants, which shape individual and collective decision-making [102]. These include knowledge, attitudes, perceived risk, self-efficacy, response efficacy, and social norms, all of which influence whether communities embrace or resist change [103,104]. SBC strategies have proven successful in fostering preventive behaviors, including increased use of ITNs [105]. However, economic and cultural barriers often limit access to preventive tools and compromise their long-term utilization. In Uganda, ITN usage remains inconsistent, with 25% of nets in households going unused due to reasons such as keeping them as a replacement (34%), not having the usual user sleep there (16%), or the nets being too old (12%) [36]. These findings suggest that financial constraints play a role, as only 1% of ITNs in Uganda are purchased, with 93% coming from mass distribution campaigns and 5% from government health centers [36]. Distance from health facilities, transportation costs, and competing family needs contribute to missed antenatal visits—where ITNs are distributed—leading to reduced ITN coverage [106]. Community leaders and health workers report that large families, low incomes, and the inability to replace worn-out nets are major obstacles to consistent ITN use [33]. Additional factors include heat, lack of visible mosquitoes, room design issues, unpleasant odors, and cultural beliefs that associate ITNs with negative effects such as impotence or attracting bedbugs [107,108].

Indoor residual spraying faces similar challenges. Despite its recognition as an effective malaria control strategy, concerns regarding insecticide toxicity, respiratory issues, unpleasant odors, and inconvenience lead to resistance among certain populations [109,110]. Socioeconomic status and education levels are positively correlated with IRS acceptance, but implementation remains largely dependent on government or NGO-led distribution programs [36]. House modifications, while effective, also present challenges. Material shortages, excessive heat, poor ventilation, and architectural constraints such as termite damage have been reported [111]. The most significant limitation, however, is high cost and low family income, making large-scale adoption difficult [112]. In Uganda, poverty has been identified as a major barrier to house modifications, with construction progressing gradually as families secure funds. Economic fluctuations, such as declines in local crop prices, further influence the feasibility of implementing protective structures [113]. Traditional mud-brick homes, common in rural areas, make these interventions even harder to sustain. Beyond financial limitations, misconceptions and cultural beliefs also hinder implementation. In some rural communities, IRS is rejected due to fears that it will drive away or anger “smaller gods” that protect the household [114]. Bed nets are sometimes believed to bring bedbugs or trap spirits inside the house, potentially causing death, particularly in the case of twins [113]. Additionally, the sale of government-distributed ITNs for income generation has been reported, undermining their intended use [108,115].

Another growing concern is insecticide resistance, particularly to pyrethroids, which threatens malaria control strategies across Africa [116]. The effectiveness of ITNs and IRS may be compromised by the selective pressure exerted by agriculture-related insecticide use, urban pollution, and widespread pesticide application [117,118]. The presence of mosquito breeding sites in irrigated rice fields and the role of maize pollen in supporting mosquito growth further illustrate the intersection between agriculture and malaria transmission [119]. As agriculture remains a cornerstone of Africa’s economy, the integration of malaria control strategies into farming policies and environmental management is crucial. Mosquitoes are also developing behavioral resistance to insecticides by changing their habits toward outdoor and daytime biting in order to avoid insecticides present indoors on walls and bed nets [120,121].

Malaria and poverty are mutually reinforcing, creating a cycle that restricts access to education, healthcare, and economic opportunities [122,123]. Uganda, a predominantly rural country, experiences significant health disparities between urban and rural areas. While urbanization is progressing, only 18% of Ugandans reside in cities [124,125], with rural populations facing higher poverty rates, limited healthcare access, and lower education levels [126–129]. With 95% of Uganda’s population living in malaria-endemic areas, disease burden remains highest in the North and Northeast regions, where health infrastructure is most limited [130].

Climate change poses additional threats to malaria control [131]. Rising temperatures, increased humidity, and changes in rainfall patterns extend mosquito breeding seasons and expand vector distribution to higher altitudes [132]. Additionally, extreme weather events displace populations, disrupt healthcare services, and exacerbate poverty, further hindering access to malaria prevention tools such as ITNs, medicines, and vaccines [133]. The evidence gathered from Uganda and Mozambique highlights the multi-layered challenges of malaria prevention, ranging from socioeconomic constraints and cultural beliefs to environmental and climate-related factors. Addressing these challenges requires integrated, long-term approaches that combine housing modifications, behavioral change strategies, and government-led malaria control programs [45,113]. Community engagement remains fundamental, as interventions must be socially and culturally acceptable to achieve sustained success [104]. The findings also reinforce the need for stronger policy support and financial investments to overcome economic barriers and scale up malaria-preventive housing solutions. Ultimately, the fight against malaria must extend beyond individual preventive measures to tackle the broader structural inequalities that perpetuate disease transmission. By integrating socioeconomic development, education, and health policies, malaria control strategies can be more effective in breaking the cycle of poverty and disease.

In Mozambique, additional challenges arise from security threats, displacement of IDPs, and geographic inaccessibility [91]. The study population included IDPs, who frequently relocate due to conflicts in the Cabo Delgado region, hindering stability and continuity of interventions. The analysis revealed that household preparedness against cholera, malaria and other climate-sensitive diseases improved significantly after the intervention [89,98]. Structural features such as the presence of improved latrines, sanitary waste disposal systems, safe water storage, and designated areas for food drying and protection from animal contamination contribute significantly to reducing environmental exposure to pathogens. These housing adaptations, when combined with social and behavior change strategies, enhance household and community preparedness, particularly in settings affected by displacement or instability [98]. The greatest improvements were seen in handwashing facilities and safe water storage, underscoring the effectiveness of SBC-based approaches in promoting health-protective behaviors, by interrupting fecal-oral transmission routes. However, some households lost access to critical preventive measures over time, emphasizing the need for sustainable infrastructure maintenance [98].

Demographic factors also played a role in intervention success. Larger, overcrowded households showed minimal improvement, whereas households with children aged 6–14 years exhibited greater adaptation, suggesting that school-based education programs can be instrumental in promoting malaria prevention strategies. Malnutrition further emerged as a critical issue, with only 52.5% of children consuming three meals per day, reinforcing the link between food insecurity and increased vulnerability to malaria and other infectious diseases [89,90].

Cholera and waterborne disease control in Mozambique does not rely solely on the presence and adoption of high-quality housing items or structural adaptations helping limit transmission by separating individuals from potential sources of infection—such as contaminated water. Equally critical is the implementation of SBC strategies, which enhance not only individual but also collective preparedness [134,135]. These strategies are particularly vital in supporting community adaptation in contexts where housing is temporary or unstable due to forced displacement, either from extreme climate events or ongoing political insecurity, or where housing is directly linked to overcrowding and consequent lack of hygiene.

Community engagement emerged as a critical determinant of intervention success, influencing both adherence and long-term sustainability. Programs that actively involve local leaders, women’s groups, and community health workers have shown greater effectiveness, as they align preventive measures with locally accepted norms and practices. Cultural acceptability is equally essential: the adaptation of interventions to local beliefs, household routines, and perceptions of risk determines the extent to which communities internalize and maintain preventive behaviors.

From a policy perspective, integrating household- and community-based interventions into national malaria and cholera control programs requires long-term political commitment, intersectoral collaboration, and consistent funding. Policymakers should support locally driven strategies, strengthen community health systems, and ensure that housing improvements, vector control, and WASH interventions are jointly planned and monitored. Such integration would foster ownership, cultural resonance, and policy coherence—key conditions for achieving sustainable reductions in vector- and water-borne diseases.

## Conclusion

Housing-based interventions, including both structural modifications and housing-related behavioral practices, play a critical role in reducing exposure to vector- and water-borne diseases such as malaria and cholera in low-income endemic countries.Two models of behavioral and structural interventions at the household level are described as low-cost, community-acceptable, and easily implementable preventive strategies against malaria and cholera. Global threats such as climate change, rapid urbanization, and globalization—largely driven by human activities—exert a disproportionate impact on vulnerable populations, particularly in the context of malaria and cholera prevention. Despite international prevention programs, low-resource populations continue to face the resurgence of certain infectious diseases such as malaria and cholera, along with significant challenges in their elimination. At the same time, the burden of these diseases exacerbates existing poverty, reinforcing a vicious cycle of poor health and economic hardship. Data from the literature indicate that relatively simple interventions, including the use of ITNs, repellent plants, regular handwashing, and improved latrines, have proven effective in reducing the transmission of infectious diseases, particularly malaria and cholera in the contexts examined. Overall, this study underscore the critical role of SBC interventions in enhancing health resilience in climate-vulnerable settings and preventing infectious diseases in resource-limited contexts. The design of health interventions should be closely integrated with an analysis of behavioral determinants, which are inherently linked to cultural, economic, and social dynamics. This multidisciplinary approach is essential for achieving sustainable public health outcomes and impactful disease prevention strategies. However, maintaining these improvements require long-term community engagement, targeted support for displaced populations, and policies that address broader structural inequalities. Strengthening these components is crucial to ensure that adaptation efforts remain both effective and equitable amid escalating global health threats.
